# An Optical Sensor for Measuring Displacement between Parallel Surfaces

**DOI:** 10.3390/s24113498

**Published:** 2024-05-29

**Authors:** Suhana Jamil Ahamed, Michael McGeehan, Keat Ghee Ong

**Affiliations:** 1 Department of Bioengineering, Knight Campus for Accelerating Scientific Impact, University of Oregon, Eugene, OR 97403, USA; sahamed@uoregon.edu (S.J.A.); mmcgeeha@uoregon.edu (M.M.); 2 Department of Human Physiology, University of Oregon, Eugene, OR 97403, USA

**Keywords:** optical sensor, shear, displacement, orthotic, prosthesis, footwear

## Abstract

An optoelectronic sensor was developed to measure the in-plane displacement between two parallel surfaces. This sensor used a photodetector, which was placed on one of the parallel surfaces, to measure the intensity of the red (R), green (G), blue (B), and white/clear (C) light spectra of a broad-spectrum light that was reflected off a color grid on the opposing surface. The in-plane displacement between these two surfaces caused a change in the reflected RGB and C light intensity, allowing the prediction of the displacement direction and magnitude by using a polynomial regression prediction algorithm to convert the RGB and C light intensity to in-plane displacement. Results from benchtop experiments showed that the sensor can achieve accurate displacement predictions with a coefficient of determination *R*^2^ > 0.97, a root mean squared error (RMSE) < 0.3 mm, and a mean absolute error (MAE) < 0.36 mm. By measuring the in-plane displacement between two surfaces, this sensor can be applied to measure the shear of a flexible layer, such as a shoe’s insole or the lining of a limb prosthesis. This sensor would allow slippage detection in wearable devices such as orthotics, prostheses, and footwear to quantify the overfitting or underfitting of these devices.

## 1. Introduction

There is an increasing need for advanced sensor technology in human–device interface research to measure mechanical shear and displacement for continuous gait evaluation [1,2]. For example, in the case of amputated limbs, it is crucial to continuously measure and monitor the residual limb-socket fitting/slip to track the level of swelling due to edema [3,4]. To address this need, researchers have developed various shear and displacement sensors based on capacitive sensing principles, resistive sensing principles, and piezoelectric materials [5,6]. However, capacitive- and resistive-based sensors can be disadvantageous for certain biomedical applications as they require high power usage [2,3], are usually heavily wired, and are thus more sensitive to electromagnetic interference and temperature and humidity fluctuation [7,8,9]. Conversely, the accuracy of piezoelectric sensors in distinguishing shear forces from other compressive forces is still inconsistent despite recent improvements in these technologies [10,11]. Compared to capacitive and resistive-based sensors, optical-based sensors would have certain advantages for medical applications because they are unaffected by electromagnetic fields and are more stable in relation to environmental factors like temperature and humidity [12,13]. Therefore, integrating optical sensors in the human-interfacing layer of medical devices and clothing, such as wearable prosthetic devices or footwear, can help improve the outcomes of medical treatments or the comfort levels of device users [12,13,14]. Furthermore, integrating shear force measurement in orthotics and prosthetics can reduce complications such as tissue ulcers and infections, as well as deep vein thrombosis in patients with diseases that are characterized by impaired sensations such as diabetic neuropathy/foot and amputated limbs [15,16,17,18].

A shear force sensor was developed and described by McGeehan and coworkers [12,13,14] that detected the red (R), green (G), blue (B), and white (C, clear/full visible spectrum) spectra of a broad-spectrum LED light that was reflected from a color grid [12,13,14]. In the study, a nine-color square grid was made up of green in the center, magenta in the four diagonal corners, and blue and red on opposite sides of the center [12]. The LED and sensor were separated from the color grid by a window (a square cutout of an elastomer layer), so at any given time, only 11% of the surface was exposed to the light [12]. When a force/displacement was applied to the color grid, the combination of the colors through the window was altered, causing changes in the reflected light spectra. A photodetector measured these changes as variations in RGB and C light intensity. The sensor’s performance was characterized by applying shear force through the elastomer and displacement under benchtop testing conditions. The sensor is scalable, low-cost, power-efficient, and less sensitive to interferences from electric and magnetic fields, temperature, and humidity. These advantages make the sensor an attractive alternative to existing technologies for a variety of applications in robotics and orthopedics.

Although the optoelectronic sensors described by McGeehan and co-workers [12] have the potential for shear force measurement in footwear and medical devices, improvements are still needed before real-world application. For example, even though McGeehan and coworkers presented results showing measurements of vertical and horizontal in-plane displacement [12], their data processing approach did not allow differentiation of the displacement/shear direction, i.e., it cannot differentiate left from right or up from down. The data processing approach in [12] could also not simultaneously measure two orthogonal directions. This paper improves upon the sensor’s design and uses a four-color square grid and a prediction tool (polynomial regression algorithm/model) to quantify multiaxial displacement in both positive and negative directions. Results from this paper provide more explicit evidence of the feasibility of this optoelectronic sensor as a practical shear-force sensor in footwear and medical devices.

## 2. Materials and Methods

### 2.1. Experiment Design

**Figure 1:**
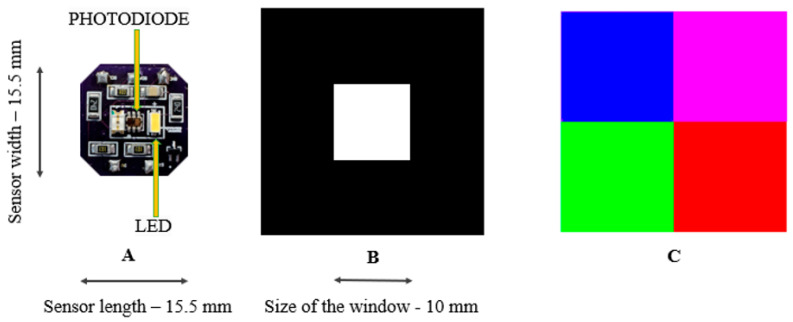
The sensor consists of a photodetector and LED on a PCB (**A**) with a square window (**B**) and a 4-cell color grid (**C**).

The sensor measured the intensity of R, G, B, and C spectra of a light emitted from an LED that was reflected off a color grid using a photodetector. The photodetector and LED were fabricated on a printed circuit board (PCB) and placed next to each other, as shown in Figure 1A. The PCB was placed under a square window, which was a square cutout of an optically opaque layer (Figure 1B). For this study, the sensor used a four-square grid with green, red, blue, and magenta (Figure 1C). The colors were selected due to the specific light sensitivity of the photodetector, which contained sensing elements with bandpass filters corresponding to red, green, blue, and clear (broad visible spectrum wavelengths). Peak responsivity of the red, green, and blue sensing elements occurs at 465 nm, 525 nm, and 615 nm, respectively. The clear channel is sensitive to wavelengths ranging from 400 to 650 nm, with peak responsivity occurring at 600 nm. In addition to the RGB colors, magenta was selected to measure diagonal shearing in the upper right quadrant because it is represented by a combination of red and blue. Shear displacement is determined from the changes in the intensity of RGB lights, which pass through the square window at a fixed optical path length. The 10 mm length of each square color matches the window in Figure 1B. The size of the square and window was kept at 10 mm × 10 mm to allow the LED and photodetector to be viewable through the window, as shown in Figure 1A.

**Figure 2:**
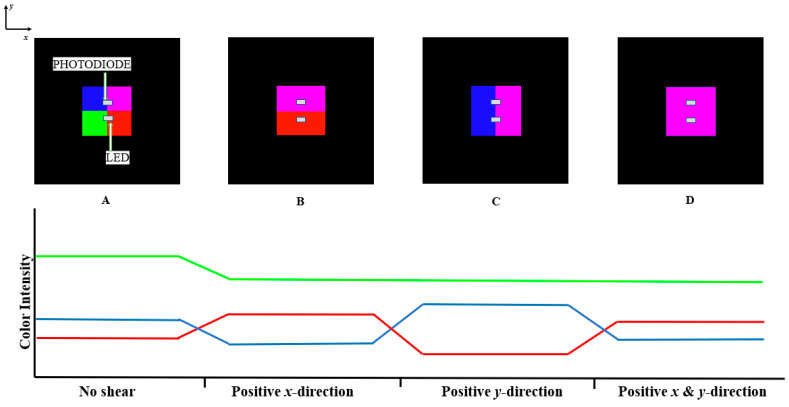
Schematic illustration of changes in the magnitude of the RGB color light intensity with displacement due to the changing color composition appearing through the window. The above graph shows the changes in green, blue, and red color light intensities and proportions of exposed color grids when the displacement occurs in the positive *x* direction (**B**), the positive *y* direction (**C**), and diagonally towards both positive *x* and *y* directions (**D**) when compared to no displacement (**A**).

When no displacement was applied to the sensor, the PCB surface and the color grid surface were fully aligned so the LED and photodetector were exposed to only 1/4 of the whole color grid with equal portions of red, green, blue, and magenta (Figure 2A). When the color grid surface is displaced in parallel to its planer axis, the intensities of the RGB and C light spectra change depending on the combination of colors at the window. As shown in Figure 2B, if the displacement is applied along the *x*-axis in the positive direction (towards the red and magenta sections), the intensity of the light increases for the red color and decreases for the green and blue colors. Similarly, if the displacement is applied along the positive *y*-direction (towards the blue and magenta sections), the blue color intensity increases, and the intensity of green and red colors decreases (Figure 2C). When the color grid surface is displaced in both *x* and *y* directions (towards the magenta section), the green color intensity decreases while the magenta color intensity increases, leading to an increase in the red and blue color intensity. This paper examines the ability of the sensor to measure displacement simultaneously along the *x*- and *y*-axes, as shown in Figure 2D.

### 2.2. Sensor Component, Design and Fabrication

The sensor consisted of three main components: a PCB (OshPark, Lake Oswego, OR, USA) with a mounted LED (Wurth Elektronik (Waldenburg, Germany), 158301240) and a photodetector (Texas Advanced Optoelectronic Solutions (Plano, TX, USA), TCS37727), a color grid, and a window layer with a square cutout that separated the PCB and color grid as shown in Figure 1. The square cutout in the window layer was 10 mm × 10 mm in size. The photodetector was 2.0 × 2.4 × 1.6 mm in size and had integrated I^2^C protocol-based, 16-bit analog-to-digital converters for data streaming with up to 400 kbit/s rates. The white (full visible spectrum) LED was 3.0 × 2.0 × 1.4 mm in size and emitted light in a 400–800 nm spectrum, with a 2000 millicandela (mcd) brightness at 20 mA. The color grid was printed on an adhesive-backed vinyl with a matte finish (Jukebox Prints Inc., Toronto, ON, Canada) and was placed on a 3D-printed fixture made up of commercially available polylactic acid (Figure 1C). The study used red, green, blue, and magenta color panels to selectively absorb or reflect corresponding colors emitted from the LED, which were then measured by the photodetector.

### 2.3. Experiment Set-Up

**Figure 3:**
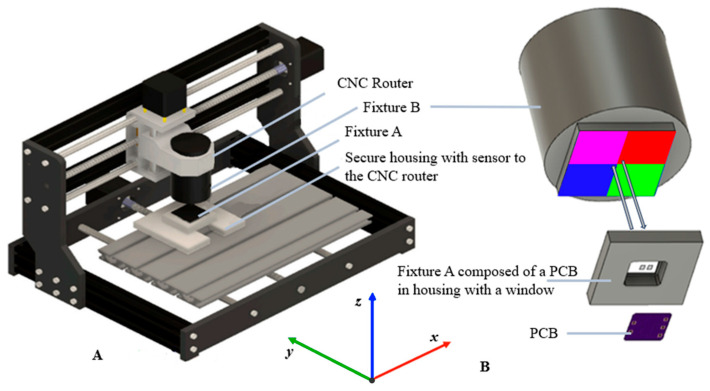
The experiment apparatus consists of a CNC machine (**A**). The PCB, color grid, and window layer are mounted to the CNC machine in a way that movements of the CNC router can cause displacement of the color grid from the PCB and the window layer (**B**).

Figure 3 demonstrates the setup used to apply controlled displacements for this study. The sensor’s measurements were recorded in response to the applied displacements through a modified computed numeric control (CNC) router (FoxAlien (Moreno Valley, CA, USA), 3018-SE V2), as shown in Figure 3. The sensor was secured on the CNC router’s machining platform in a 3D-printed fixture (Fixture A in Figure 3). The color grid was affixed at the bottom of Fixture B and was secured in the CNC spindle bracket to provide controlled displacement with the CNC’s stepper motors (NEMA 17). The use of CNC apparatus also ensured that Fixture A and Fixture B were precisely parallel with each other during the experiments. When the color grid layer and PCB layer were aligned, the LED and photodetector were each exposed to 1/4 of each color grid. The CNC router was operated through a Python script to provide displacement and record data.

### 2.4. Data Collection

To examine the effect of tilting (non-parallel) between the color grid layer and PCB layer, the PCB layer was tilted at a 5° angle from the color grid layer, followed by measuring the intensity of the RGB and C light spectra. It was found that the changes in light intensity were less than 5% for a small tilt angle of 5°. To demonstrate the precision and reliability of the sensor measurements, two data sets were collected by varying the displacement between the color grid and the PCB/window layer in the *x* and *y* planes with 1 mm and 0.1 mm spatial resolutions. These two spatial resolutions were selected to demonstrate the precision and reliability of the sensor measurements for applications of different scales. Higher resolutions (e.g., 0.01 mm) were not investigated due to the limitations of the CNC machine. In addition, resolutions larger than 1 mm were not examined since these resolutions were too low for practical use in humans.

**Figure 4:**
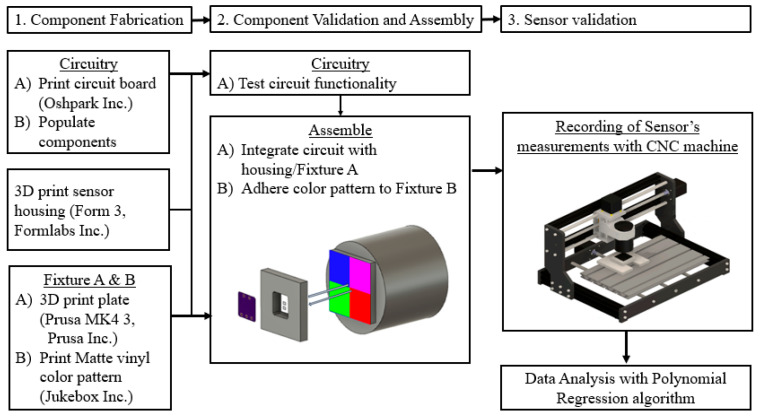
Flow diagram summarizing the sensor’s design, fabrication, and characterization processes (Formlabs Inc., Somerville, MA, USA; Prusa Inc., Prague, Czech Republic).

For the 1 mm resolution, the displacement occurred at 1 mm increments for a range of −5 mm to +5 mm in the *x* and *y* directions, as illustrated in Figure 3. Similarly, for the 0.1 mm resolution, the displacement occurred at 0.1 mm increments for a range of ±1.5 mm in the *x* and *y* directions (Figure 3). Before the experiment, a total of ten data points were streamed at each position for both data sets to confirm that no variability was found in those ten measurements. Therefore, only one static measurement of R, G, B, and C intensity was collected in the experiment. For the 1 mm resolution measurement, sensor responses were recorded at 121 positions across the −5 mm and 5 mm space in both the *x* and *y* directions. The recording at these 121 positions was repeated six times, resulting in 726 data points for each light color and displacement position for the 1 mm resolution set. Likewise, for the 0.1 mm resolution, sensor responses were recorded at 961 positions with six repeats at each position, forming a total of 5766 data points for each light color. To observe the best performance of the sensor, all data were collected under no ambient light. Figure 4 summarizes the process of sensor fabrication architecture, fabrication, operation principles, and experimental set-up.

#### 2.4.1. Data Acquisition and Analysis

**Figure 5:**
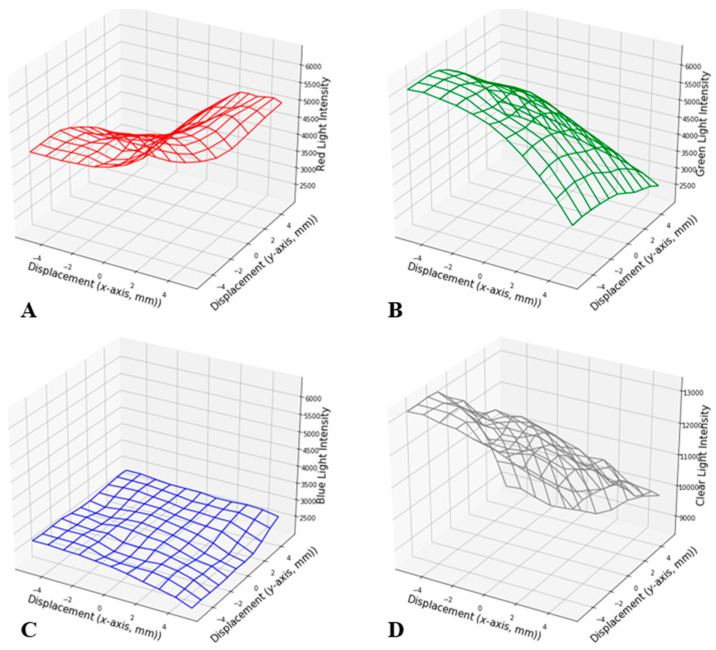
Measured red color light intensity (**A**), green color light intensity (**B**), blue color light intensity (**C**), and clear color light intensity (**D**) as a function of in-plane displacement between the PCB surface and color grid surface at 1 mm spatial resolution.

**Figure 6:**
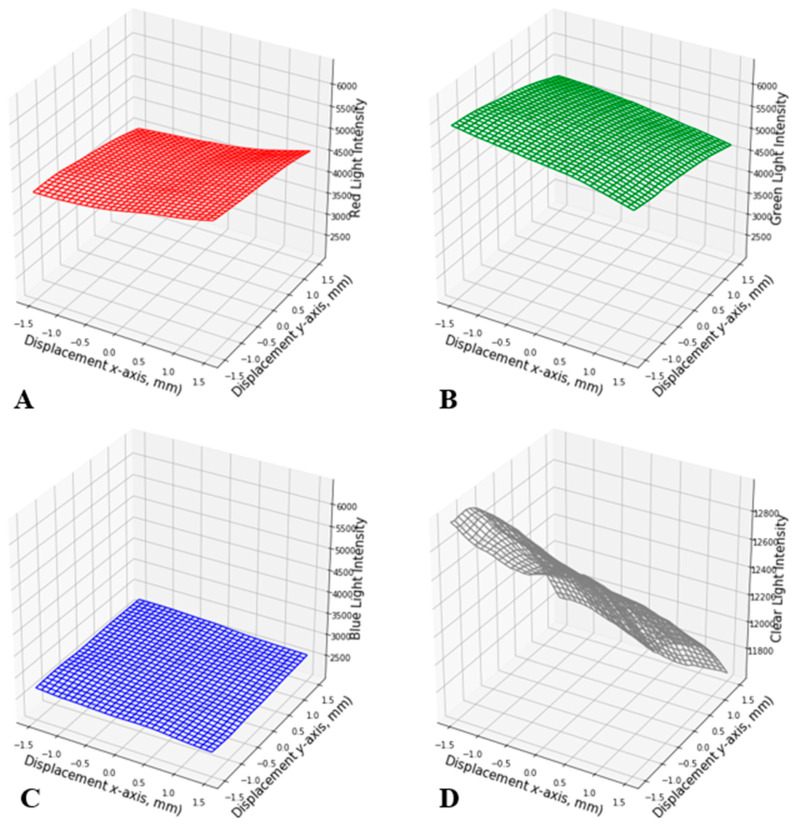
Measured red color light intensity (**A**), green color light intensity (**B**), blue color light intensity (**C**), and clear color light intensity (**D**) as a function of in-plane displacement between the PCB surface and color grid surface at 0.1 mm spatial resolution.

The average of six data points was calculated for each position for both 1 mm and 0.1 mm resolution sets and plotted in Figure 5 and Figure 6, respectively. In both figures, the intensity of RGB and C lights was plotted on the *z*-axis as a function of the *x* and *y* displacement between the color grid surface and the PCB surface. The intensity of each color was measured as a unitless 16-bit number, where 0 represents no light and 65,535 is the brightest measurement before saturation. Overall, light intensities of RGB and C spectra corresponded with the color grid at the window. For example, for the 1 mm resolution set, there was a decrease in green color intensity as the displacement increased along the *x* and *y* axes (Figure 5B). Meanwhile, there was an increase in red color intensity with increasing *x*-axis displacement (Figure 5A) and an increase in blue color intensity with increasing *y*-axis displacement (Figure 5C). It is interesting to note the similar gradual increase in red color intensity and negligible change in blue color intensity when the combined *x* and *y* displacements (diagonal direction) were performed, implying that magenta has an unequal ratio of red and blue colors. However, the green color exhibited a consistent decrease in its intensity along any directional displacement (Figure 5A,C). Similarly, the 0.1 mm resolution set exhibited a similar trend as the 1 mm resolution set, with a decrease in green color intensity with increasing *x* and *y* displacement (Figure 6B), and an increase in red and blue color intensity along the *x* direction (Figure 6A) and *y* direction (Figure 6C), respectively.

In Figure 5 and Figure 6, some abrupt dips of light intensity were observed at multiple coordinates. This may be due to the LED’s asymmetric positioning, the mismatches between the color on the grid surface, and the light wavelengths the LED used for its corresponding color. Figure 5D and Figure 6D demonstrate the 3D representation of the clear light intensity at each position for 1 mm and 0.1 mm resolution sets, respectively, which is a summation of the R, G, and B color light intensities. However, these patterns are neither linear nor distinct. Thus, a fitting algorithm was used on the R, G, and B color intensities as input to generate displacement coordinates as output (*x*, *y*). The following section focuses on the algorithm used for this study, i.e., the polynomial regression model in machine learning.

#### 2.4.2. Polynomial Regression Model

In previous studies, the recorded applied displacement data from the CNC device and the R, G, B, and C light intensities (outputs from the sensor) were used to design a prediction model [12]. The output values from the sensor (R, G, B, and C light intensities) were used as independent variables to predict whether the applied displacement occurred in the *x* or *y* coordinate [12]. In previous studies [12], data were organized as a classification set to predict either the *x* or *y* direction outcomes. In this study, the outputs from the sensor (R, G, B, and C intensity values) were used as independent variables to predict the absolute *x* and *y* coordinates, allowing the determination of both the shear displacement’s magnitude and direction (positive and negative values in either *x* or *y*). Each data set used in this study contained continuous data; this meant that numerical values, instead of discrete values, were used for the classification dataset. To approximate the relationship of two outcomes (*x* and *y* coordinates) with multiple data features (R, G, B, and C), the polynomial regression model was deemed fit to identify the non-linear pattern.

**Figure 7:**
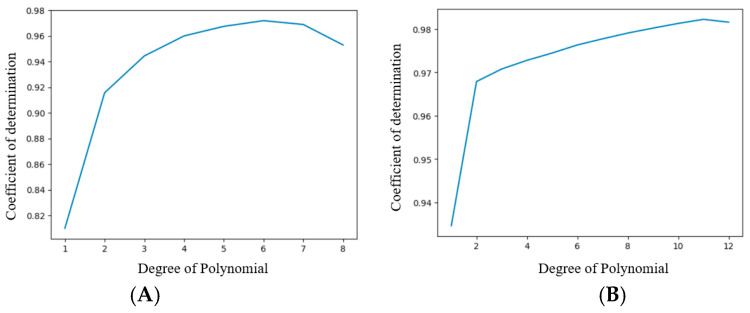
*R*^2^ at different degrees of order for 1 mm resolution set (**A**) and 0.1 mm resolution set (**B**). The graphs demonstrate the highest *R*^2^ value of 0.972 at the degree of the polynomial 6 for the 1 mm resolution set (**A**) and the highest *R*^2^ value of 0.98 at the degree of the polynomial 11 for the 0.1 mm resolution set (**B**).

Polynomial regression was used here as it allows non-linear relationships between variables to be captured by introducing higher-order polynomial terms (e.g., quadratic, and cubic) and provides a more accurate representation of the complex data patterns. However, the algorithm also carries a high risk of overfitting at higher orders, thus it is crucial to select and design the model carefully. To obtain a fitting model, this regression analysis requires splitting the data into a training set, test set, and validation set. The most significant chunk of data is set aside for training, where the underlying relationships and patterns among the variables are analyzed. Validation data are used to fine-tune the parameters and assess the performance during the training process. Finally, test data are used to evaluate the performance of the trained model. Data splitting is performed by randomly selecting the data points for each process, often tuned by a random number (referred to as the parameter *random_state*). The typical workflow involves iteratively training the model on the training set, tuning parameters (degree of polynomial) using the validation set, and assessing its performance on the test set. The coefficient of determination (*R*^2^), root mean squared error (RMSE), and mean absolute error (MAE) are used to evaluate the accuracy of the continuous dataset. Figure 7A and Figure 7B show the model’s performance with increasing order of the polynomial for data with resolutions of 1 mm and 0.1 mm, respectively. The RMSE and the MAE are common metrics by which to evaluate the performance of a regression model. A lower MAE value indicates that the model’s prediction is closer to the actual values, being a better fit. The MAE value does not square the differences, making it less sensitive to extreme outliers compared to the RMSE value.

#### 2.4.3. Model Fitting

For this study, different data split ratios and randomized values were tested to avoid algorithm bias using Python programming and sci-kit (SK) learn libraries. In addition, the algorithm’s predictive performance was assessed on different degree orders with different split ratios and *random_state* numbers. This paper presents the algorithm’s results with a 70%, 15%, and 15% data split ratio among the training, test, and validation sets, respectively, with a *random_state* value of 62 for both 1 mm and 0.1 mm resolution sets. Two separate polynomial models were designed for the 1 mm and 0.1 mm resolution sets. Figure 7A and Figure 7B plot the performance assessment of the models using *R*^2^ at the different degrees of order for the 1 mm and 0.1 mm resolution sets, respectively. In the figures, the highest *R*^2^ value occurs at the degrees of polynomial 6 and 11 for the 1 mm and 0.1 mm resolution sets, respectively, before decreasing due to overfitting.

## 3. Results

### 3.1. Performance Assessment

The polynomial regression model predicted the *x* and *y* displacement coordinates based on the R, G, B, and C light intensity measurements. These parameters were concluded based on cross-validation of the results from the validation set applied to the test set. For the 1 mm resolution set with 6 degrees of polynomial, the *R*^2^, RMSE, and MAE values were 0.972, 0.285, and 0.359, respectively. For the 0.1 mm resolution set with 11 degrees of polynomial, the *R*^2^, RMSE, and MAE values were 0.980, 0.016, and 0.081, respectively.

Table 1 shows the value of all metrics used to determine the accuracy of the fitted data and the degree of the polynomial for both 1 mm and 0.1 mm resolution sets. The observed values of *R*^2^ for both resolutions ensure the data fit well. Subsequently, RMSE and MAE for both resolutions ensure high data accuracy.

The visualization of model predictions for *x* and *y* coordinates was carried out separately for each resolution set by plotting a scatter graph of actual and fitted values. The scatter graphs were plotted by juxtaposing the actual values on the *x*-axis against the predicted value on the *y*-axis. A straight line representing the fitted model with an *R*^2^ value of 1 was drawn on the same graph, which helps visualize the variance of predicted values from the actual values.

### 3.2. Predicted Values vs. Actual Values

**Figure 8:**
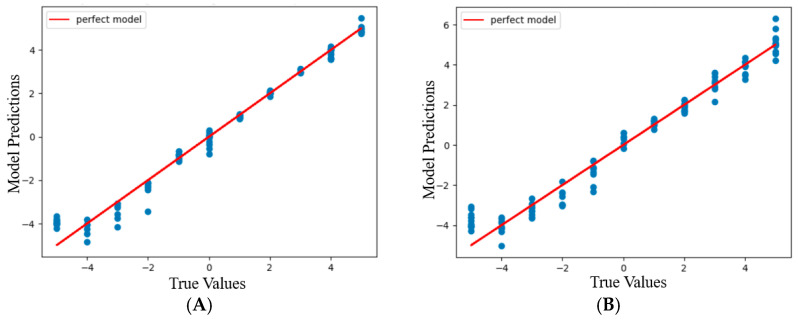
Graphs showing the model prediction and true values of scaled *x* displacements (**A**) and *y* displacements (**B**) at the degree of the polynomial 6 for the 1 mm resolution set.

**Figure 9:**
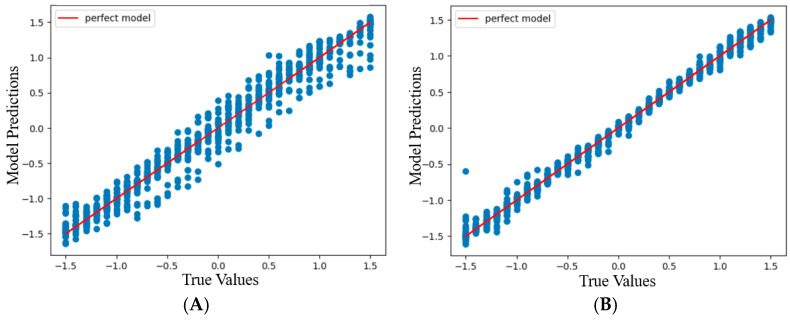
Graphs showing the model prediction and true values of scaled *x* displacements (**A**) and *y* displacements (**B**) at the degree of the polynomial 11 for the 0.1 mm resolution set.

In Figure 8 and Figure 9, most data points from the prediction appear very close to the straight line. This explains the high *R*^2^ value and the low RMSE and MAE values. However, for both data sets, a few data points are further away from the straight line. These points appear in the −5 to −2 mm range for the 1 mm resolution set (Figure 8A) and almost through the entire range of *x*-axis displacement for the 0.1 mm resolution set (Figure 9A). On the contrary, these points are seen through the −5 to −2 mm range for the 0.1 mm resolution set (Figure 8B) and almost through the entire range of the *y*-axis displacement for the 1 mm resolution set (Figure 9B). These outliers may be due to the abrupt dips in R, G, B, and C light intensity data, which were possibly caused by the LED’s asymmetric positioning and/or the mismatches between the color on the grid surface and the corresponding wavelengths used by LED. The abrupt dips led to a large variation in the predicted values of the *x* and *y* coordinates. Despite a high number of outliers present in the 0.1 mm resolution set in comparison to the 1 mm resolution set, the *R*^2^ value of the 0.1 mm resolution set is higher. This may possibly be due to the larger size of the dataset and a smaller number of outliers along the *y*-axis displacement (Figure 9B).

## 4. Discussion

This paper is part of an ongoing project to develop a reliable, efficient, and cost-effective commercially available sensor for biomedical applications [12]. The current study demonstrates the multiaxial response that can determine the direction of displacement compared to a previously reported optical sensor. Although this study demonstrates further advances toward the development of a human–device interface tool, there are limitations to the sensor before ready for real-world application. A future step towards the goal is for the sensor to predict angular displacement in addition to displacement/shear, allowing this technology to measure the combined linear, multi-directional, and rotational values of force and displacement. Another aspect of development is to minimize the device’s power consumption and size, which are critical for applications such as monitoring human gait biomechanics using in-shoe sensors.

Integrating shear force measurement in shoes/orthotics/prosthetics can be an effective tool for rehabilitation in conditions such as diabetic neuropathy/foot and amputated limbs. Such patients often experience clinical complications like swelling, sensory loss, and the death of skin tissue [19,20,21]. Furthermore, orthotic footwear and prosthetics with improper fitting can cause ulcers and tissue scarring, aggravating clinical complications, and sometimes even necessitating the re-amputation of the limb [19,20,21]. The sensor presented here can help prevent such a grave prognosis by monitoring the shear force and displacement in the skin layer and the shoe sole’s material, advancing the scope of human–device interface usage for an effective clinical management and treatment protocol. A future goal is to enclose the sensor between the layers of the shoe’s insole, so the color grid pattern is on the most superficial layer of the insole to interact with plantar skin. Similarly, the ongoing project is progressing on strategies to enclose the sensor into the socket of the prosthesis, as described in related published articles [12,13,14].

## 5. Conclusions

This paper presented the design, fabrication, and validation of a lightweight and low-cost sensor based on the optical interaction of an LED, photodetector, and color grid. Benchtop experiments show the sensor has high accuracy and sensitivity to predict multiaxial coordinates of a displacement based on polynomial regression modeling of R, G, B, and C light intensities obtained by a photodetector. The highly accurate prediction results from this study, combined with the sensor’s miniaturized design, support its use for various biomedical and clinical applications. The goal of this research project is to design and enhance the performance of insole shoes with embedded sensors that can be used to monitor and assess the shear forces along with kinetic and kinematic changes over time in the human population.

## Figures and Tables

**Table 1:** Summarizes the metric values for 1 mm and 0.1 mm resolution sets.

Metric	Dataset (1 mm)	Dataset (0.1 mm)
*R* ^2^	0.972	0.980
RMSE	0.285	0.016
MAE	0.359	0.081
Degree of polynomial	6	11

## Data Availability

Data will be made available upon reasonable request to the authors.
